# Targeting IL-11R/EZH2 signaling axis as a therapeutic strategy for osteosarcoma lung metastases

**DOI:** 10.1007/s12672-024-01056-3

**Published:** 2024-06-18

**Authors:** Eswaran Devarajan, R. Eric Davis, Hannah C. Beird, Wei-Lien Wang, V. Behrana Jensen, Arumugam Jayakumar, Cheuk Hong Leung, Heather Y. Lin, Chia-Chin Wu, Stephanie A. Ihezie, Jen-Wei Tsai, P. Andrew Futreal, Valerae O. Lewis

**Affiliations:** 1 Department of Orthopaedic Oncology, The University of Texas MD Anderson Cancer Center, 1515 Holcombe Boulevard, Unit 1448, Houston, TX 77030 USA; 2 Department of Lymphoma and Myeloma, The University of Texas MD Anderson Cancer Center, Houston, TX USA; 3 Department of Genomic Medicine, The University of Texas MD Anderson Cancer Center, Houston, TX USA; 4 Department of Pathology, The University of Texas MD Anderson Cancer Center, Houston, TX USA; 5 Department of Veterinary Medicine and Surgery, The University of Texas MD Anderson Cancer Center, Houston, TX USA; 6 Department of Experimental Therapeutics, The University of Texas MD Anderson Cancer Center, Houston, TX USA; 7 Department of Biostatistics, The University of Texas MD Anderson Cancer Center, Houston, TX USA; 8 McGovern Medical School, The University of Texas Health Science Center at Houston, Houston, TX USA; 9 Department of Anatomic Pathology, E-Da Hospital, Kaohsiung, Taiwan

**Keywords:** Osteosarcoma, PRC2 complex, EZH2 inhibitors, H3K27me3, Metastasis

## Abstract

Lung metastases are the primary cause of death for osteosarcoma (OS) patients. We recently validated interleukin-11 receptor α (IL-11Rα) as a molecular target for the inhibition of OS lung metastases. Since there is no clinically approved antibody against this receptor, we sought to identify downstream targets that mediate the effects of IL-11Rα signaling. We used shRNA to deplete IL-11Rα from OS cells; as a complementary approach, we added IL-11 exogenously to OS cells. The resulting changes in gene expression identified EZH2 as a downstream candidate. This was confirmed by knockdown of IL-11Rα in OS cells, which led to increased expression of genes repressed by histone methyltransferase EZH2, including members of the WNT pathway, a known target pathway of EZH2. Exogenous IL-11 increased the global levels of histone H3 lysine 27 trimethylation, evidence of EZH2 activation. Treatment with the EZH2 inhibitor GSK126 significantly reduced in vitro proliferation and increased cell-cycle arrest and apoptosis, which were partially mediated through the WNT pathway. In vivo*,* treatment of an orthotopic nude mouse model of OS with GSK126 inhibited lung metastatic growth and prolonged survival. In addition, significantly shorter recurrence-free survival was seen in OS patients with high levels of EZH2 in their primary tumors (P < .05). This suggests that IL-11Rα promotes OS lung metastasis via activation of EZH2. Thus, blocking EZH2 activity may be an effective strategy for inhibiting OS lung metastasis and improving prognosis.

**Supplementary Information:**

The online version contains supplementary material available at 10.1007/s12672-024-01056-3.

## Introduction

Osteosarcoma (OS) is the most common primary malignant bone tumor in children and young adults [1]. Although chemotherapy has improved the 5-year survival, the prognosis for patients with OS has not changed over the past 40 years. Local disease is often controlled with chemotherapy and surgical resection, but many patients still develop pulmonary metastases, compromising their survival [2]. Patients with relapsed disease only have a 20% survival rate [3]. Systemic chemotherapy treatment is associated with significant morbidity, including renal failure, cardiac compromise and neuropathy [4, 5]. Alternative noncytotoxic targeted therapeutics, including tyrosine kinase inhibitors and insulin-like growth factor-1 inhibitors, have shown limited effectiveness against human OS or are associated with intolerable adverse effects [6–10]. The best alternative therapeutic options would be efficacious agents with specific targets that cause minimal collateral systemic damage.

Previously, we demonstrated that interleukin-11 receptor α (IL-11Rα) is overexpressed in both OS primary tumors and lung metastases, and short hairpin RNA (shRNA)-mediated knockdown of IL-11Rα can inhibit the development of lung metastasis in orthotopic models of OS [11]. Using phage displaying a peptide with high-affinity binding to IL-11Rα, a well-known cytokine signaling pathway that controls osteoclast development, we found strong and highly-specific accumulation of phage in orthotopic OS tumors in mice [12]. Metastatic OS has a higher expression of IL-11Rα than in primary OS samples, normal tibia, or lung tissue samples and silencing of IL-11Rα by shRNA inhibited lung metastasis [12].

However, there is no clinically approved antibody against IL-11Rα, so we sought to identify the downstream targets that mediate its effects. One such candidate is the enhancer of zeste homolog 2 (EZH2), the catalytic methyltransferase subunit of the multimeric polycomb repressive complex 2 (PRC2), which includes SUZ12 and EED [13]. The PRC2 complex mediates histone H3 lysine 27 trimethylation (H3K27me3), which results in epigenetic silencing of genes involved in proliferation and invasion. Since the effects of H3K27me3 are tissue-, context-, and disease-specific, EZH2 can function as either a tumor suppressor or an oncogene. Inhibiting EZH2 in T-cell acute lymphoblastic leukemia activates NOTCH1 [14]. In diffuse large B-cell lymphoma and follicular lymphoma, recurrent point mutations in the catalytic SET domain (Y641) of EZH2 increase H3K27me3 activity, repressing the expression of tumor suppressor genes that mediate differentiation, cell-cycle inhibition, and apoptosis [15–17]. In prostate cancer, breast cancer, myeloma, and OS, high EZH2 levels are associated with tumor invasiveness and metastasis [13]. Currently, EZH2 is the only component of the PRC2 complex for which small-molecule inhibitors are being tested or have already been approved for certain indications. Given the availability and potential therapeutic effects of EZH2 inhibitors and the dire need for new OS treatment options, we conducted preclinical studies to explore the potential therapeutic benefit of EZH2 inhibition on osteosarcoma progression and the development/suppression of pulmonary metastatic disease .

## Materials and methods

### Cell culture

The human OS cell lines KRIB (CVCL_AU05), SJSA1 (CVCL_1697)*,* CCH-OS-D (CVCL_XG67), Saos-2 (CVCL_0548**)**, MG63 (CVCL_0426) and HOS (CVCL_0312) were either obtained from the American Type Culture Collection (ATCC) or were internally established cells were cultured as recommended [11]. These cell lines were authenticated by short tandem repeat profiling at the Cytogenetics and Cell Authentication Core at UT MDACC.

### shRNA knockdown and gene expression profiling by microarray

Knockdown studies using shRNA were performed with KRIB cells as previously described [11] using an IL-11Rα–specific shRNA and a control GIPZ lentiviral shRNA (GE Healthcare). Total RNA was extracted from knockdown cells using a *mir*Vana miRNA Isolation Kit (Life Technologies). Microarray analysis was performed with Illumina HumanHT-12 v4 Expression BeadChip array. Array data were processed as described previously using quantile normalization, "flooring" of data to the detection threshold, and elimination of unreliable probes [18].

### Immunohistochemical staining (IHC) of OS samples for IL-11 and H3K27me3

Tissue microarrays (TMA) containing formalin-fixed, paraffin-embedded (FFPE) specimens of recurrent and metastatic OS tumors from 200 patients were built according to National Cancer Institute guidelines. The protocol was approved by the institutional ethics, biohazards, and Institutional Review Board at UT MDACC. IHC was performed with unstained 4- μM-thick slices, decalcified TMA, and using an anti-human IL-11 antibody (polyclonal, 1:100; LifeSpan BioSciences) and an anti-human H3K27me3 antibody (clone C36B11, 1:200; Cell Signaling Technology) with a Leica Bond-III autostainer (Leica Biosystems).

### Real-time reverse-transcription polymerase chain reaction analysis

An AgPath-ID One-Step RT-PCR Kit (Applied Biosystems/Life Technologies) was used with TaqMan probes on StepOnePlus PCR System (Applied Biosystems) to quantify *EZH2* expression in IL-11Rα and EZH2 shRNA-treated OS cell lines (SJSA1 and KRIB). *EZH2, SUZ12,* and *EED* expression levels were quantified using 10 ng of total RNA. Cyclophilin A was used as an endogenous control.

### EZH2 knockdown and lentivirus infection

First, 293 T cells were transfected with EZH2–specific (GE Healthcare) and control GIPZ lentiviral shRNA plasmids (Dharmacon), together with packaging vectors, following the manufacturer's instructions. At 48–72 h post-transfection, viral particle-containing supernatants were collected, filtered through 0.45-µm-pore filters, and used to infect SJSA1 and KRIB cells in the presence of 8 µg/mL polybrene (Sigma-Aldrich). After another 72 h, shRNA-transduced GFP-expressing cells were separated by cell sorting with a BD FACSAria instrument (BD Biosciences) according to the manufacturer's instruction as previously described (11), and EZH2 silencing was confirmed by qPCR and immunoblotting.

### Immunohistochemical staining of EZH2

FFPE samples of primary tumors (n = 18) and lung metastases (n = 27) were identified from the UT MDACC OS database and hospital tumor registry and were used to build a TMA. Antigen retrieval via heating with EDTA (pH 8.0; Zymed Laboratories) was followed by biotin and protein blocking (Dako). Expression of EZH2 was evaluated using a rabbit anti-EZH2 antibody (clone AC22; Cell Signaling Technology) diluted at 1:15 (vol/vol) and incubated for 45 min, followed by development using an LSAB + Kit (Dako). Staining was evaluated as described previously [11]. Recurrence-free survival was defined as the period from the time of surgery to the time of first relapse or death or to the time of last contact. The Kaplan–Meier method and log-rank test estimated the recurrence-free survival distributions. All studies were performed in accordance with the guidelines of the Institutional Review Board.

### Addition of exogenous IL-11 to CCH-OS-D cells and histone isolation

On the first day, 3 × 10^3^ CCH-OS-D cells were plated into a petri dish. The next day, they were treated with recombinant human IL-11 (R&D Systems, 218-IL) at increasing concentrations (100, 200, 300 ng) and EZH2 shRNA SJSA1 and KRIB cells for (200 ng) for 48 h. The histone extraction protocol from Abcam was used for histone isolation (ab1134760. A dish of untreated cells was used as a control for quantitative PCR (qPCR) and Western blot analyses.

### H3K27 methylation status with GSK126 treatment

SJSA1, KRIB, and CCH-OS-D Cells were seeded into 100 mm tissue culture plates in the appropriate cell culture media 24 h before treatment. Cells were then exposed to 0.1% DMSO or varying concentrations of GSK126 (range 2, 1, and 0.5 μM) for 24 h, then histones were isolated and used for western blotting.

### Western blot analysis and antibodies and immunoprecipitation

SJSA1, KRIB, HOS, and CCH-OS-D OS cells were lysed as previously described [11]. Primary antibodies against EZH2 (Cell Signaling Technology, 5246), EED (GeneTex, Inc., GTX628007), H3K27me3 (Millipore Sigma, 07–449), total histone (Abcam, ab1791) and β-actin (Cell Signaling Technology, 12620) were performed. Enhanced chemiluminescence detection was performed using the SuperSignal West Dura Extended Duration Kit (Thermo Fisher Scientific). For immunoprecipitation, CCH-OS-D and SJSA1 cells were treated with IL-11(200 and 400 ng). Treated cells were lysed with RIPA buffer plus protease inhibitors, and the EZH2 antibodies used for immunoprecipitation (Millipore AC22 CS-203193).

### In vivo* orthotopic model of OS*

Mouse experiments were conducted in compliance with the policies and procedures of the UT MD Anderson Cancer Center Institutional Animal Care and Use Committee. Two-week-old male nude mice were purchased from the National Cancer Institute. OS cells were injected into the right tibia (2 × 10^4^ cells/mouse) of the mice [11, 12]. Before and throughout all imaging and surgical procedures, mice were anesthetized as previously described. [11] Mice will be amputated when tumor burden reached 1.5 cm. Subsequently mice will be randomized into treatment groups and monitored by imaging. When mice are moribund with weight loss and difficulty breathing mice will be euthanized. In all experiments tumor size did not exceed 1.5 cm. All studies were performed in accordance with the guidelines of Institutional Animal Care and Use Committee at UT MD Anderson Cancer Center.

### Leg amputation

Mice underwent either stifle or hip disarticulation once their primary tumors reached at least 1.5 cm^3^ in volume, approximately 21 days after injection. Post-operative care consisted of subcutaneous saline and heat packs. Mice were monitored until they fully recovered from anesthesia.

### Preclinical therapeutic protocols

Once primary tumors reached a volume of 4 to 5 mm^3^, mice were randomized to treatment groups. One group received treatment with GSK126 intraperitoneally at 0.1 mL (150 mg per 20 g of body weight) in 20% Captisol (cyclodextrin vehicle) every 2 days for 28 days similar to the middle dosage used by McCabe et al. [19]. Control mice received Captisol on the same schedule (300 mg/kg once every 2 days for 4 weeks). Mice were weighed, and their primary tumor growth was measured using calipers twice weekly. Lung metastases were monitored using luciferase imaging. Mice were euthanized at day 48, and their lungs were harvested and processed into FFPE samples for histopathological analysis.

### Proliferation assay

Cell proliferation was measured with the use of a colorimetric assay in 96-well plates with 2-(4-iodophenyl)-3-(4-nitrophenyl)-5-(2,4-disulfophenyl)-2H-tetrazolium monosodium salt (WST-1 reagent; Roche). One thousand cells per well were plated onto a 96-well plate in triplicate. The next day, cells were treated with increasing concentrations of GSK126 (0.0625–20.0000 µM) for 48 h. WST-1 was added to the cells and incubated for 2 h. Cell proliferation was measured at 450 nm using a microplate reader (DTX880; Beckman Coulter), and the cytotoxicity of GSK126 was expressed as the percentage of viable cells. Half-maximal inhibitory concentrations (IC_50_ values) were calculated using Prism software (version 6.0; GraphPad Software).

### Apoptotic cell death and flow cytometric cell cycle analysis

For three independent replicates, OS cells were starved for 24 h in serum-free medium and then cultured overnight in complete growth medium. A panel of OS cells was treated with vehicle or GSK126 (1 and 2 µM) for 96 h, cells were collected and stained with annexin V and propidium iodide for the apoptosis assay and DAPI for the cell-cycle assay. Cells treated with 1 and 2 µM GSK126 were analyzed using flow cytometry (BD Biosciences) and FlowJo software.

### RNA sequencing

Coding regions were captured using a SureSelectXT RNA Direct Library Preparation Kit (Agilent Technologies) and sequenced using the Illumina HiSeq 2000 system to generate 75-bp paired-end reads. Preprocessing was performed as previously described [20]. Differential expression analysis between GSK126-treated cells (both 1 µm and 2 µm) and control cells (DMSO) was performed using the limma [21]. Pathway analyses of genes were performed using gene set enrichment analysis [22].

### Ex vivo* bioluminescent imaging*

Mice were implanted with luciferase-expressing SJSA1 variants and then injected subcutaneously with 15 mg/mL luciferin potassium salt in phosphate-buffered saline at 150 mg/kg body weight. Imaging was performed as previously described [11].

## Results

### Gene expression changes in IL-11Rα–knockdown cells

**Fig. 1:**
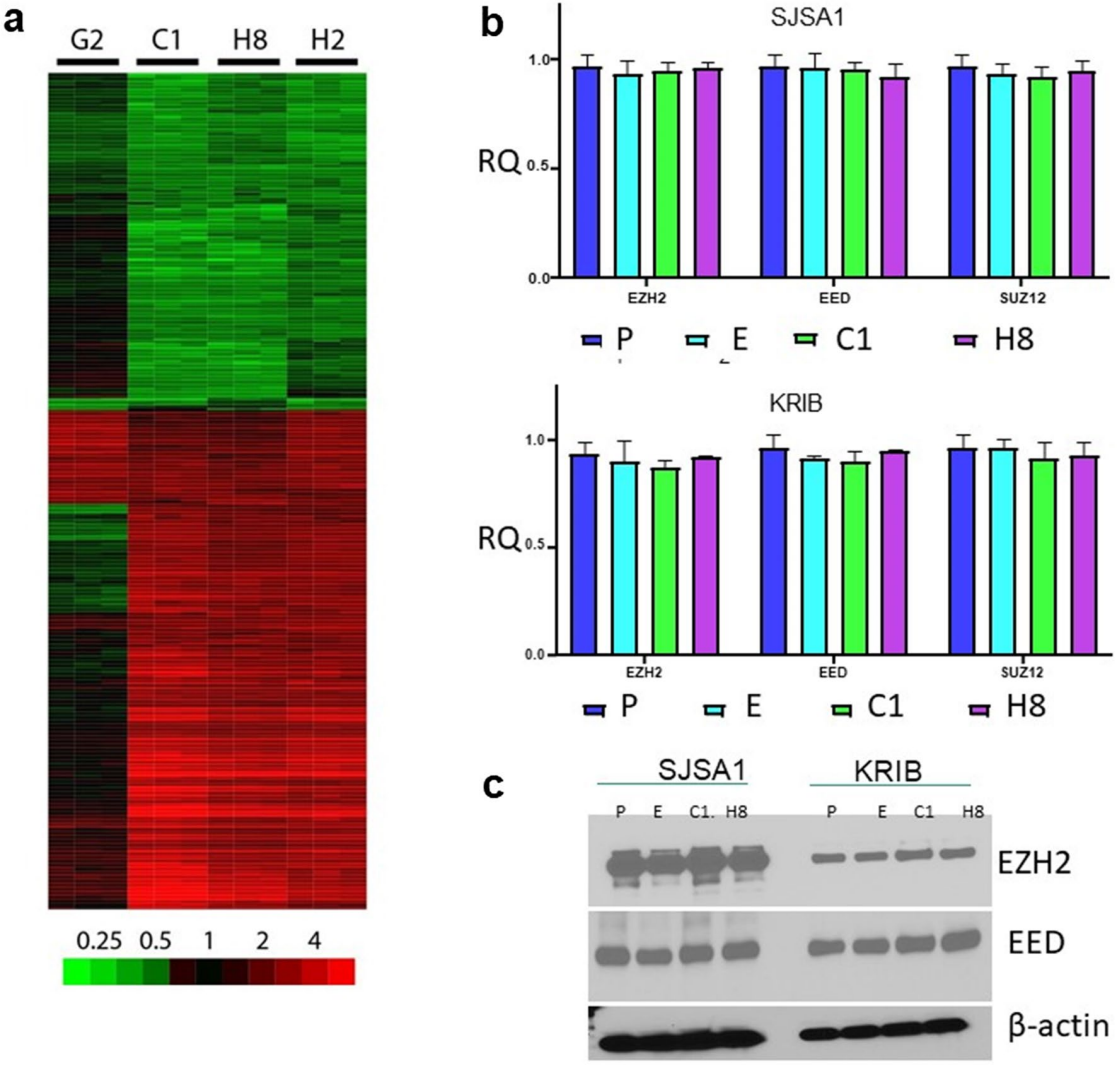
Gene expression changes after IL-11Rα knockdown or IL-11 exogenous expression in the OS cells. **a**, Four shRNA sequences (G2, C1, H8, and H2) targeting IL-11Rα were stably expressed in KRIB cells. Gene expression was measured by microarray. Processed and log2-transformed gene expression data were compared to that of control cells. The heat map shows subtracted values for the gene probes in which values for shRNA sequences C1, H8, and H2 differed on average by at least twofold from those in the control cells. The color bar shows the fold-change difference from the values in the control cells. **b**, Quantitative reverse-transcription PCR analysis of the mRNA expression levels of the PRC2 complex members *EZH2*, *EED*, and *SUZ12* in SJSA1 (upper panel) and KRIB cells (lower panel). P—parental cells, E—empty vector, C1—IL-11Rα knockdown via shRNA C1, H8—IL-11Rα knockdown via shRNA H8, RQ—relative quantity. **c** Immunoblot of EZH2 and EED protein levels in SJSA1 and KRIB cells. β-actin served as a loading control. P—parental cells, E—empty vector, C1—IL-11Rα knockdown via shRNA C1, H8—IL-11Rα knockdown via shRNA H8, RQ—relative quantity.

We examined the effects of IL-11Rα knockdown in OS cell line KRIB by using transcriptome microarrays. For cells transduced and selected with lentivirus expressing shRNA sequences targeting IL-11Rα, comparison of gene expression to that of untransduced parental cells showed differences that were highly reproducible across technical replicates and similar for 3 of the shRNA sequences (C1, H2, and H8; Fig. 1a), which reduced IL-11Rα expression fourfold. A fourth shRNA sequence (G2), which induced only a twofold reduction, produced dissimilar and less-pronounced changes and was not further considered. For the 3 shRNA sequences with similar reductions in IL-11Rα, comparison to control cells identified 270 genes (of 11,153 total) that were upregulated and 216 downregulated by at least twofold (Supplementary Table S1).

Gene set enrichment analysis with genes ranked according to fold-change differences between IL-11Rα knockdown (shRNA sequences C1, H2, and H8) and control replicates (Supplementary Table S2) was performed. Several of the most significantly upregulated gene sets following IL-11Rα knockdown were related to the PRC2 chromatin remodeling complex and histone H3 epigenetic modifications. For instance, the set with the second greatest significance was LU_EZH2_TARGETS_UP (*P* < 0.001; false discovery rate [FDR], q < 10^–3^), which includes genes that were upregulated in SKOV3ip1 ovarian cancer cells upon knockdown of EZH2 [23]. The third most significant gene set was MARTENS_TERTINOIN_RESPONSE_UP (*P* < 0.001; FDR, q < 10^–3^), which contains genes that were upregulated in NB4 acute promyelocytic leukemia cells in response to treatment with all-trans retinoic acid, which alters H3 acetylation. The top 20 gene sets also included those with genes whose promoters contain H3K4me3 (MIKKELSEN_IPS_LCP_WITH_H3K4ME3: *P* < 0.001; FDR, q < 0.1) or H3K27me3 (MIKKELSEN_MEF_HCP_WITH_H3K27ME3: *P* < 0.001; FDR, q < 0.15). In addition, genes downregulated in TIG3 fibroblasts upon knockdown of EED were enriched (PRC2_EED_UP.V1_DN: *P* < 0.001; FDR, q < 0.05). These data suggest that IL-11Rα knockdown affected genes related to PRC2 and genes whose promoters rely on H3 modifications for controlling their expression. Other observations include the Wnt/β-catenin pathway, which was significantly deregulated upon IL-11Rα knockdown (WNT_SIGNALING: *P* < 0.01; FDR, q < 0.1, Supplementary Table S2), with the most dramatic expression level changes in *WNT7B* and *TCF20* (Supplementary Figure S1). Several microRNAs were upregulated in cells after the knockdown of IL-11Rα, such as miR-488 and miR-7-3HG (Supplementary Figure S2).

### IL-11 increases PRC2 and H3K27me3 in OS Cells

**Fig. 2:**
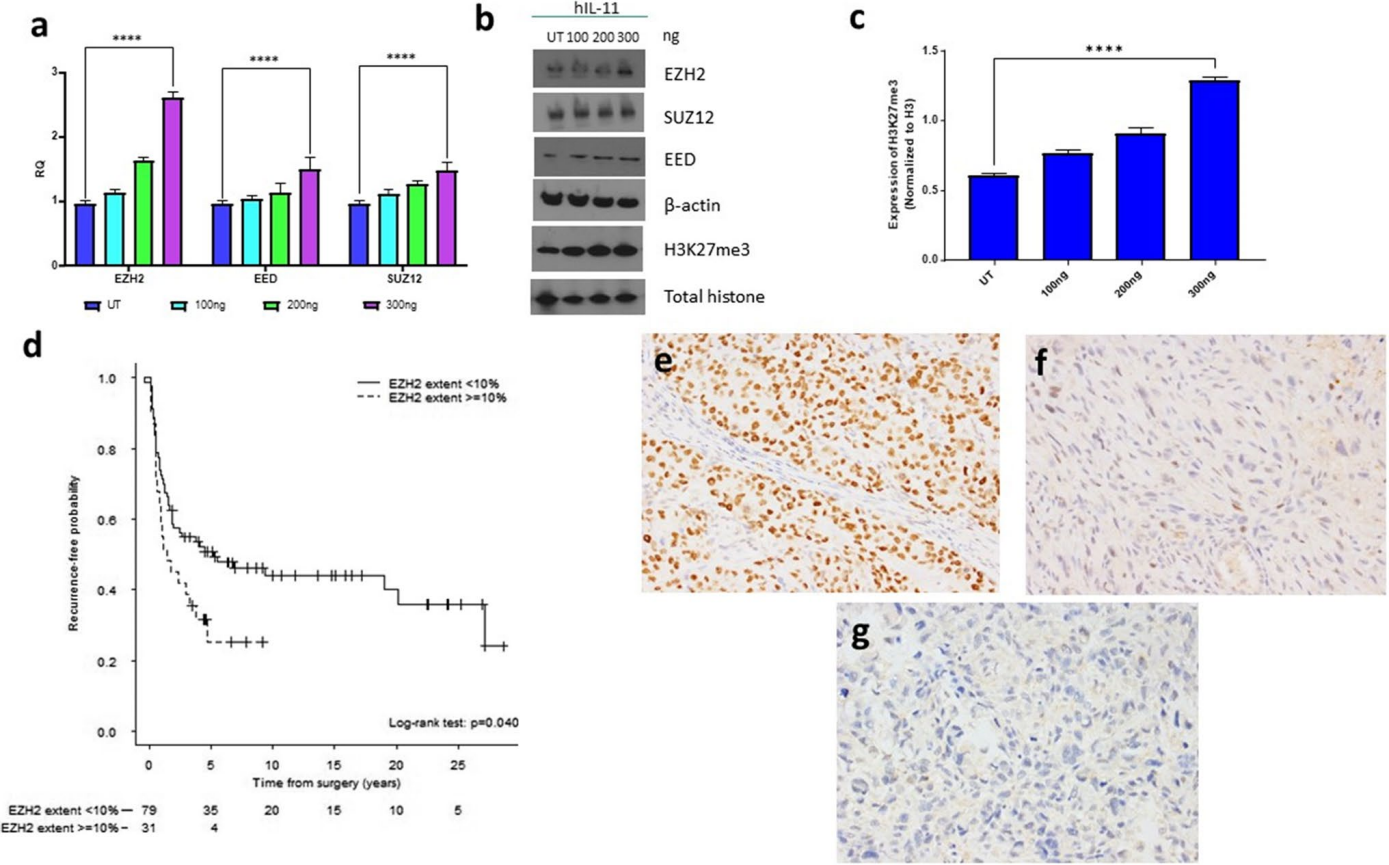
Exogenous addition of human IL-11 to OS cells enhances PRC2 activity. Cells were treated with human recombinant IL-11 (hIL-11; 100, 200, and 300 ng) for 1 h and analyzed by **a**, qPCR for *EZH2*, *EED*, and *SUZ12* values, normalized to PPIA, and **b**, immunoblotting for H3K27 methylation status and expression of PRC2 complex member proteins (EZH2, SUZ12, and EED). Total histone and β-actin were used as loading controls. **c** Densitometry measurements of H3K27me3 levels normalized to total histone H3 for each dose of hIL-11. UT – untreated, ****P < 0.0001. **d** Kaplan–Meier curves showing probability of recurrence-free survival by extent of EZH2 staining. **e**–**g** The extent of positive EZH2 staining (percentage of positively stained nuclei) was scored on TMAs composed of 200 formalin-fixed, paraffin-embedded, decalcified human OS samples (141 primary and 59 metastatic). **e** Strong diffuse nuclear labeling. **f** Weak nuclear labeling. **g** Negative. Magnification 200X

Our microarray data suggested that IL-11Rα knockdown affects PRC2 activity and may alter epigenetic programming. To survey the expression of PRC2 complex members, we performed qPCR and Western blotting on IL-11Rα shRNA-knockdown OS cells. Knockdown of IL-11Rα had no effect on either RNA (Fig. 1b) or protein levels (Fig. 1c) of the PRC2 components EZH2, EED, and SUZ12 in KRIB or SJSA1 cells. To determine the effect of IL-11 activation on PRC2, we added exogenous human IL-11 to CCH-OS-D cells, which do not express IL-11 but do express IL-11Rα [11]. As shown in Fig. 2, the addition of exogenous human IL-11 increased *EZH2* RNA levels (Fig. 2a) in a dose-dependent manner. EZH2 and H3K27me3 protein levels (Fig. 2b, c) were also increased similarly without any changes in the levels of total histone H3. Therefore, active IL-11 signaling affects PRC2 levels and activity.

### High levels of EZH2 expression are associated with worse recurrence-free survival

To determine the prognostic value of EZH2, we performed immunohistochemical staining for EZH2 using primary tumors obtained from OS patients with known outcomes. Patients whose tumors had greater than 10% or more tumor cells with positive nuclear staining for EZH2 had worse recurrence-free survival rates than did those with lower than 10% positively stained tumor cells, which is consistent with what has been observed by others (Fig. 2d–g) [24–26].

**Fig. 3:**
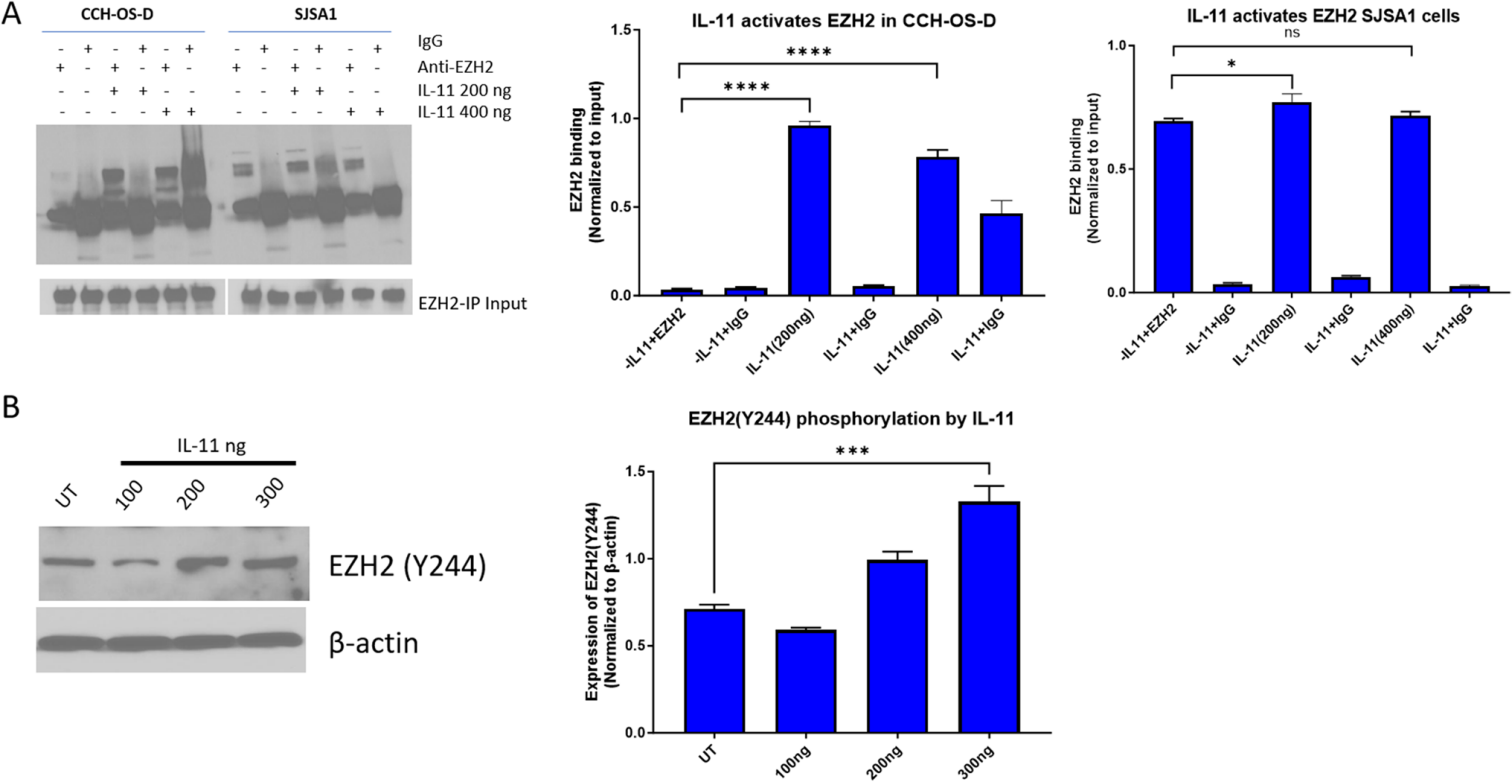
IL-11 activates EZH2 OS cells **a** Immunoprecipitation assay of EZH2 in CCH-OS-D and SJSA1 cells treated with human IL11 (200 or 400 ng) for 24 h. **b** CCH-OS-D cells were treated with human IL-11 for 48 h and then used for immunoblot analysis of phosphorylated EZH2-Y244. β-actin was used as loading control

To address whether EZH2 might be a target of the IL-11/IL-Rα pathway, we treated the CCH-OS-D and SJSA1 OS cell lines with IL-11 (200 and 400 ng) for 24 h and immunoprecipitated with EZH2 antibody or IgG control. Western blotting showed enrichment of EZH2 upon IL-11 treatment in both cell lines (Fig. 3a); the effects of exogenous IL-11 addition were more pronounced in CCH-OS-D cells, consistent with the previous observation that only SJSA1 cells express endogenous IL-11(11). Next, we investigated whether IL-11 addition can lead to phosphorylation of EZH2 at Y244, which is required for activation of EZH2. Treatment of CCH-OS-D cells, which do not express IL-11, with human IL-11 produced increased EZH2 Y244 phosphorylation in a dose-dependent manner (Fig. 3b). These data indicate that the IL-11/IL-11Rα pathway may play a role in EZH2 activation in OS.

To determine whether there was evidence for this relationship in OS patients, we conducted immunohistochemical staining for H3K27me3 and IL-11 using a TMA containing local recurrent and metastatic OS specimens (Supplementary Figures S3 and S4). We found that 38 of 89 (43%) recurrent and 30 of 48 (63%) metastatic OS samples had strong IL-11 staining. For H3K27me3, 62 of 96 (65%) recurrent and 34 of 61 (56%) metastatic OS samples had moderate to strong staining (Supplementary Table S3). There was a significant positive correlation between the extents of staining for IL-11 and H3K27me3 (the percentages of positively stained nuclei) among locally recurrent tumors (*P* < 0.05) (Supplementary Tables S4 and S5). No other significant associations or correlations were observed between the 2 markers.

## Inhibitory effect of EZH2 on IL-11 mediated H3K27me3 activation

**Fig. 4:**
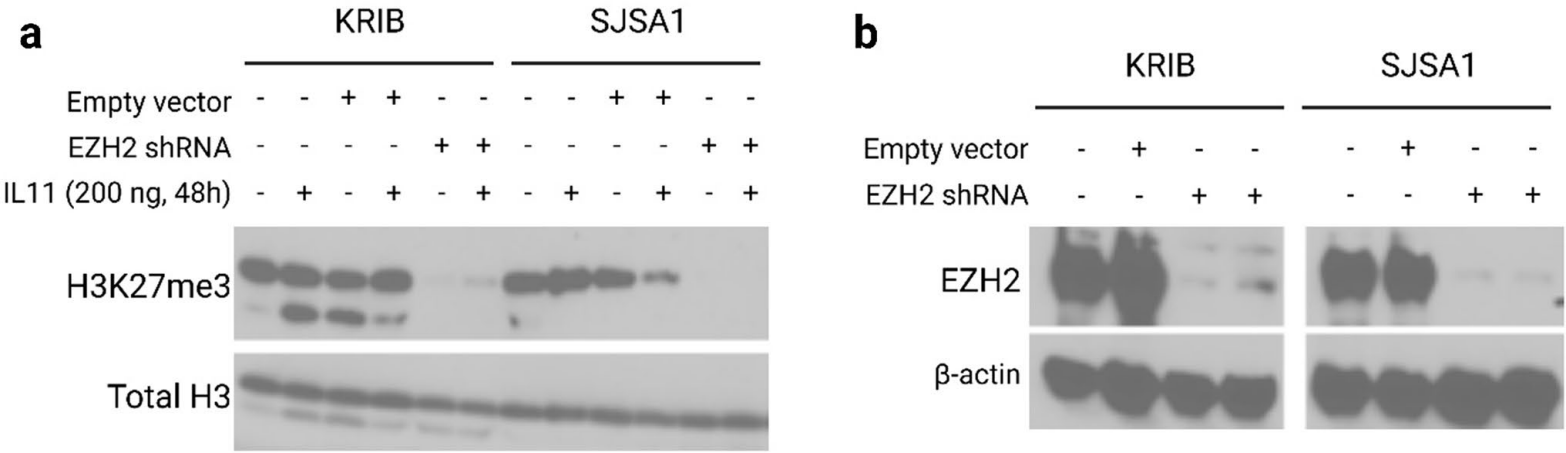
IL-11 mediates H3K27 trimethylation. **a** KRIB and SJSA1, EZH2shRNA and vector control cells were treated with human IL-11 for 48 h, Cells were harvested and used for immunoblot with H3K27 antibody. Total H3 was used as a control. **b** Immunoblot analysis of EZH2 knockdown and vector control in KRIB and SJSA1 cells. β-actin was used as a loading control

It has been established that a high level of EZH2 and activation of H3K27me3 is found in many cancer types and confers a significantly poor prognosis. Down-regulation of EZH2 suppresses the growth and invasion in cancer cells, so we wanted to explore the relationship between EZH2 and H3K27me3 in the IL11/IL-11Rα pathway. To investigate that we generated SJSA1 and KRIB EZH2 knockdown cells using shRNA lentivirus. Knockdown efficiency was analyzed by qPCR and western blotting. Further, we treated SJSA1 and KRIB EZH2 shRNA cells with human IL-11 (200 ng) for 48 h before isolating their histones. Western blotting showed that EZH2 knockdown markedly decreased H3K27me3 in both lines, at baseline and after IL-11 treatment (Fig. 4). These data demonstrate that IL11 alters the methylation of H3K27 that is mediated by EZH2.

### GSK126 decreases the viability of OS cells

**Fig. 5:**
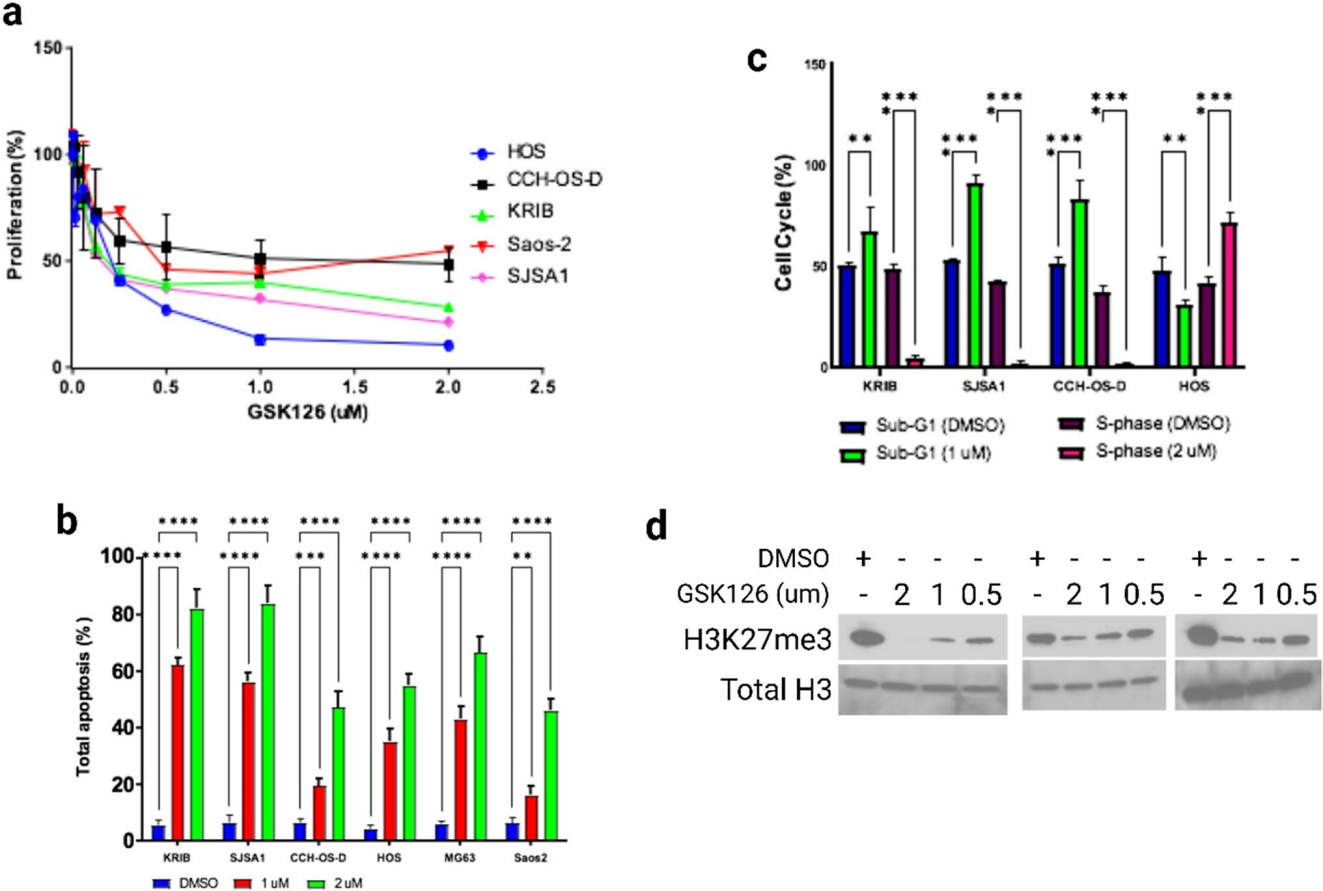
GSK126 inhibits proliferation and H3K27me3 of OS cells. **a** OS cell lines were treated for 48 h with GSK126, and their proliferation was assessed using the WST-1 assay. GSK126 inhibited the proliferation of KRIB, HOS, and SJSA-1 cells. Half-maximal inhibitory concentrations (IC_50_) values are available in Table S4. **b** and **c** Treatment with GSK126 induced apoptosis and cell-cycle arrest in OS cells. **b** Results of annexin V–fluorescein isothiocyanate/propidium iodide analysis of the indicated OS cell lines after 24 h of treatment with GSK126 (*P* < .01). The error bars represent standard error of the mean. c, Results of flow cytometric analysis of the cell-cycle progression in OS cell lines after 24 h of treatment with GSK126. GSK126 induced sub-G1 arrest in KRIB, SJSA1, and CCH-OS-D cells and S-phase arrest in HOS cells (*P* < .05). The error bars represent standard error of the mean **P < 0.01; ***P < 0.001; ****P < 0.0001. **d** Evaluation of H3K27me3 in SJSA1, KRIB and CCH-OS-D cell lines following GSK126 treatment for 72 h. Total histone H3 is shown as a loading control

We assessed the possibility of using EZH2 inhibition as a therapeutic option for primary and metastatic OS. We first tested the effect of the S-adenosyl-L-methionine–competitive, small-molecule EZH2 inhibitor GSK126 on the viability of 5 OS cell lines. Proliferation was inhibited in a dose-dependent manner for 4 (KRIB, CCH-OS-D, SJSA1 and HOS) of the 5 OS cell lines tested, of which 3 lines (KRIB, HOS, and SJSA1) were particularly sensitive to GSK126 (Fig. 5a, Supplemental Table S6). The IC_50_ values for all lines ranged from 0.1987 to 1.045 µM.

### GSK126 induces apoptosis and cell-cycle arrest in OS cells

OS cell lines that were treated with GSK126 displayed a high proportion of apoptotic cells (Fig. 5b). Increasing the concentration of GSK126 from 1 to 2 µM enhanced apoptosis 4.5-fold (*P* ≤ 0.01). Flow cytometric analysis demonstrated a significant elevation in the number of G1-phase cells and a decline in the number of S-phase cells upon GSK126 treatment (Fig. 5c), indicating decreased proliferation, although HOS cells were an exception. The addition of GSK126 to SJSA1, KRIB AND CCH-OS-D cell lines also decreased trimethylation of H3K27 (Fig. 5d). Therefore, GSK126 compromises the cell viability and H3K27me3 in OS cells, some being more sensitive than others.

**Fig. 6:**
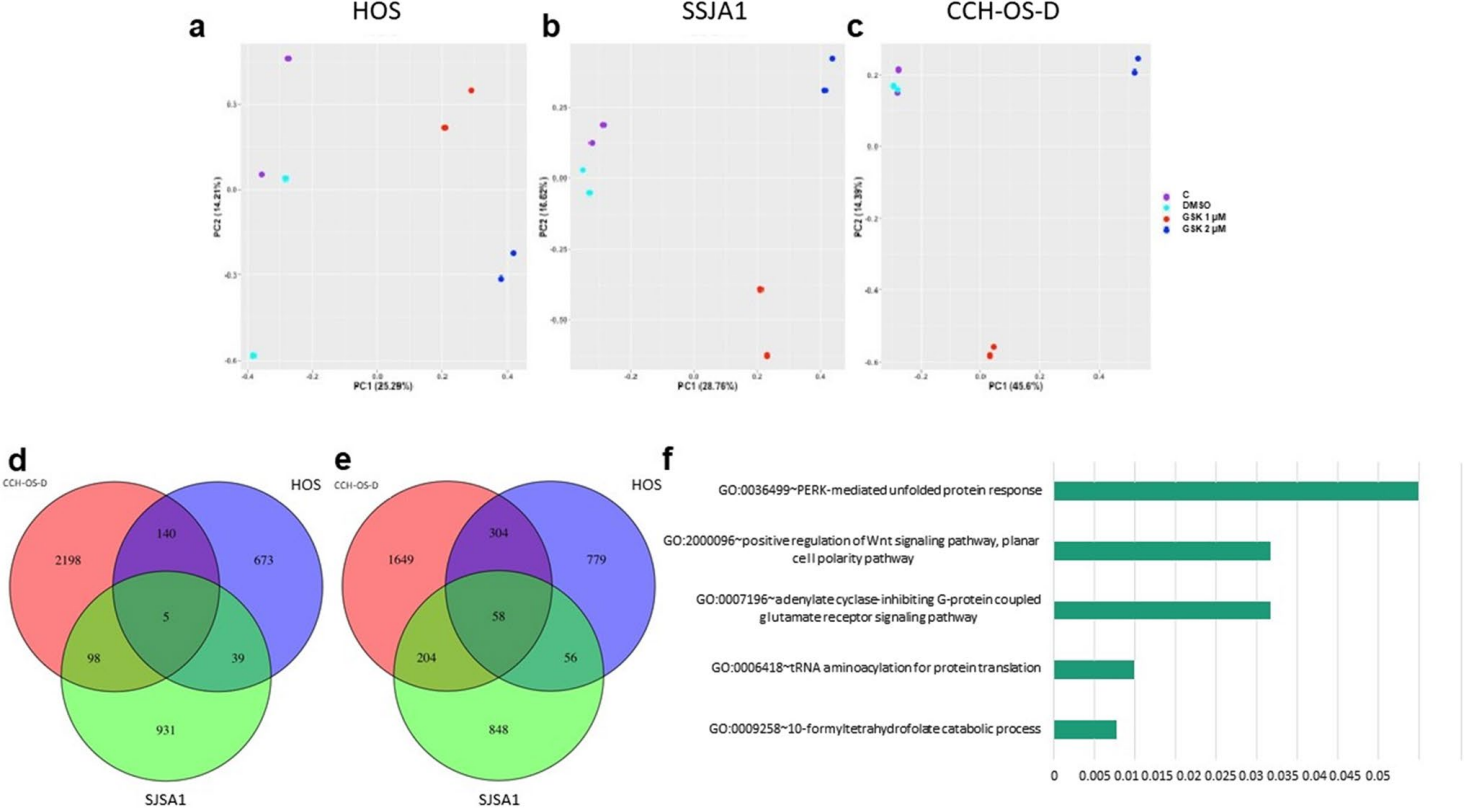
RNA sequencing of OS cells treated with GSK126. **a**-**c** Principal component analysis of RNA expression levels of genes for GSK126-sensitive cell lines HOS and SJSA1 (**a** and **b**) and the GSK126-resistant cell line CCH-OS-D **c**. subjected to no treatment (control, C), treatment with dimethyl sulfoxide (DMSO, vehicle), or treatment with GSK126 at 1 or 2 µM. **d** and **e** Venn diagrams showing the intersection of significantly upregulated and downregulated genes (upon GSK126 treatment, both 1 or 2 µM, as compared to control and DMSO) of the 3 cell lines. **f** Bar plot of the Gene Ontology enriched pathways

We performed RNA sequencing on OS cells before and after treatment with GSK126 at 1 and 2 µM. HOS and SJSA1 cells were the most sensitive to treatment, whereas CCH-OS-D cells were less sensitive (Fig. 5a). Principal component analyses demonstrated distinguishable, likely dose-dependent gene expression changes in these cells after treatment (Fig. 6a–c). We found 39 upregulated and 56 downregulated genes in the GSK126-sensitive cells (Fig. 6d, e). Enriched pathways included **(**Fig. 6f**)** neuroactive ligand-receptor interactions (*P* < 0.01), transfer RNA-related pathways (*P* < 0.05), and Wnt signaling (*P* < 0.05) (Supplementary Table S7). The most highly ranking Wnt pathway members in this enrichment were *NKD1* and *RSPO3*, both of which had reduced levels in the sensitive cell lines after GSK126 treatment.

### GSK126 inhibits lung metastasis in an orthotopic model of OS

**Fig. 7:**
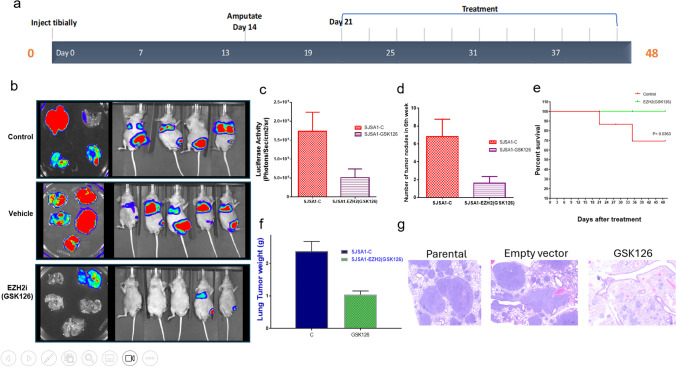
Inhibition of OS lung metastases by GSK126 in vivo. **a** Luciferase imaging of mice injected with SJSA1 cells comparing day 3, when primary tumors had developed, and day 21, after primary tumors had been removed by amputation but before treatment initiation. The bottom panels shows luciferase imaging and lung tumors for mice treated with control-, vehicle (cyclodextrin)-, and EZH2 inhibitor (EZH2i; GSK126) by day 48 after the start of the regimen. **b** Quantification of luciferase activity. **c** number of tumor nodules in control-treated versus GSK126-treated mice. **d** Survival curves for all mice. **e** Weight of lungs in control- and GSK126-treated mice. **F** Representative images of H&E staining of explanted lungs treated with Vehicle and GSK126. Scale bar: 100 μm

In our hands, only two OS cell lines are readily metastatic: SJSA1 and KRIB. However, KRIB cells are transfected with Kirsten murine sarcoma virus Ki-Ras, and thus the mechanism of metastasis may not represent what occurs in patients. SJSA1 cells were therefore used to examine the in vivo effects of GSK126 on lung metastases. Luciferase-expressing SJSA1 cells were injected into the tibiae of nude mice. The affected limb was amputated when primary tumors reached 1.5 cm^3^ in volume. GSK126 was given at 150 mg per 20 g of body weight every 2 days for 28 days. At the end of 48 days, luciferase imaging revealed significant inhibition in the growth of pulmonary metastases (Fig. 7a, b), fewer lung nodules (Fig. 7c), lower tumor weights (Fig. 7e) and higher rate of survival in treated mice 48 days after treatment (*P* ≤ 0.0363) (Fig. 7d), in the treated mice GSK126 compared to the control-treated or vehicle-treated mice (*P* ≤ 0.01). This result was further confirmed by histological analysis of hematoxylin and eosin (H&E)-stained formalin-fixed, paraffin-embedded (FFPE) tissue sections (Fig. 7f).

## Discussion

IL-11 is a cytokine produced by normal osteoblasts and presumably regulates bone remodeling in a manner similar to that of IL-6 [27]. This pathway appears to be intact in OS, as stimulation of some OS cells with osteotropic agents such as transforming growth factor-β and prostaglandins increases the levels of IL-11 [27]. Previously, we observed that IL-11/IL-11Rα signaling in OS cells promotes lung metastasis in an orthotopic model [12]. In the present study, we compared OS cell transcriptomes before and after the knockdown of IL-11Rα. Several miRNAs were increased, including mIR-488 and mIR-103A1. Induction of miR-488 reduces the proliferation, invasion, and epithelial-mesenchymal transition of the OS cell line U2OS [28]. Elevated levels of miR-103A1 promote invasion and epithelial-mesenchymal transition in multiple OS lines [29]. These microRNAs may also promote metastasis.

PRC2 complexes were responsible for much of the downstream changes in expression. Our observation supports that IL-11 stimulation increases expression of PRC2 complex members, activation of EZH2 by phosphorylation of Y244, and levels of H3K27me3. Knocking down EZH2 prevents H3K27me3 by IL-11 and EZH2 levels are independent of IL11R levels. Furthermore, OS patients have a significant positive association between IL-11 and H3K27me3 immunostaining. Therefore, IL-11 activates EZH2 to methylate H3K27.

Although other mechanisms of increasing EZH2 expression exist, our study is the first to demonstrate that IL-11/IL-11Rα signaling can activate EZH2. Transcription factors such as Myc in prostate cancer cells [30] and the EWS-FLI1 fusion protein in Ewing sarcoma cells [31] can bind to the *EZH2* promoter and enhance its expression. In addition to transcription factors, several microRNAs and long noncoding RNAs (lncRNAs) directly regulate *EZH2* expression [13, 32–34].

Our study confirms that EZH2 protein levels are associated with aggressive tumor behavior and poor outcomes in OS patients [24, 25, 35]. In vitro data demonstrate that small interfering RNA knockdown of *EZH2* inhibits OS cell growth, proliferation, and invasion, and decreases cancer stem cell functions. In vivo data showed fewer lung metastases. EZH2 levels have also been shown to be associated with metastasis of pediatric rhabdomyosarcoma, Ewing sarcoma, and synovial sarcoma [26, 36]. It remains to be seen whether EZH2 promotes metastasis in the same manner across tumor types.

The molecular mechanism by which EZH2 enhances metastasis is still unclear. One consistent downstream effect of EZH2 noted in other cancer types is the perturbation of the Wnt pathway. We too observed this in both IL-11Rα knockdown cells and cells treated with GSK126. In colon cancer, hepatocellular carcinoma, precancerous mammary epithelial lesions, and breast cancer cells, EZH2 binds to β-catenin and activates Wnt signaling [37–40]. The relationship may also be in a feedback loop where the Wnt/β-catenin pathway may positively regulate PRC2 and affect invasiveness [41].

EZH2 has been targeted pharmacologically in various tumor types with the small-molecule inhibitor GSK126 [42], a highly selective EZH2 inhibitor that competes with S-adenosyl-L-methionine binding [19]. In agreement with the results of these previous studies, we found that GSK126 repressed the growth of several OS cell lines, with IC_50_ values ranging from 0.1987 to 1.045 µM. For SJSA1, KRIB, and CCHOS-D cells, the addition of GSK126 prevented trimethylation of H3K27. Thus, our study further supports the therapeutic use of EZH2 inhibition in OS patients. Multiple types of EZH2 inhibitors appear to have similar effects in OS cells. For instance, treatment with the EZH2 inhibitor GSK343 inhibited Saos2 cell viability by attenuating cell-cycle progression and promoting apoptosis [43]. GSK343 inhibited the expression of EZH2 and its targets c-Myc and H3K27me3 and inhibited the expression of FUSE-binding protein 1 in these cells [43]. Likewise, treatment with the EZH2 inhibitor DZNep decreased the expression of EZH2 and H3K27me3 in U2OS and Saos2 OS cells [25]. We also observe a decrease in H3K27me3 after treating with GSK126. All five OS lines showed dose-dependent increases in apoptosis and G1 cell cycle arrest. However, they differed in their proliferation sensitivities. These differences may indicate that resistance mechanisms could vary among patients, with some having more proliferative abilities than others.

Our results further demonstrated that IL-11 and IL-11Rα act upstream of the PRC2 complex and H3K27me3. This relationship allowed us to demonstrate that either silencing IL-11Rα or debilitating the PRC2 complex through EZH2 inhibition can reduce primary tumor growth and prevent lung metastatic growth in vivo. Reduced lung tumor growth was also verified by H&E staining. Therefore, inhibiting EZH2 activity is a viable therapeutic proxy for inhibiting IL-11 signaling. Furthermore, our work, and that of others, demonstrated that the poor prognosis of OS patients is associated with high EZH2 expression and activity. Currently, the only trial using EZH2 inhibition in OS patients is the Pediatric MATCH trial (NCT03155620), which requires the presence of an EZH2 mutation for eligibility. We found no point mutations in *EZH2* in the OS patient samples we examined [20], and none have been reported in HOS, Saos2, or SJSA1 cells, for which sequencing data are publicly available [44]. Therefore, the requirement of an EZH2 mutation in OS may be overly restrictive in that EZH2 can be active in OS cases without gain-of-function mutations.

In summary, our data suggest that EZH2 is downstream of the IL-11R pathway. Blocking EZH2 activity results in a reduction of osteosarcoma lung metastases. The findings here suggest that EZH2 inhibitors may be a viable therapeutic option for the treatment of  OS patients.

### Supplementary Information

Supplementary material 1.

## Data Availability

The microarray data used to support the findings of this study were deposited in the Gene expression Omnibus repository (GSE 191215) on December 19, 2021 [45].
