# Supplementary material for: Targeting IL-11R/EZH2 signaling axis as a therapeutic strategy for osteosarcoma lung metastases
Source: Discov Oncol. 2024 Jun 18;15:232. doi: 10.1007/s12672-024-01056-3 (PMC11183017; doi:10.1007/s12672-024-01056-3)
Supplement: Supplementary file 1 — Supplementary material 1. [file 12672_2024_1056_MOESM1_ESM.zip › 12672_2024_1056_MOESM1_ESM/New folder/Suppl. Fig.1.pptx]

## Slide 1
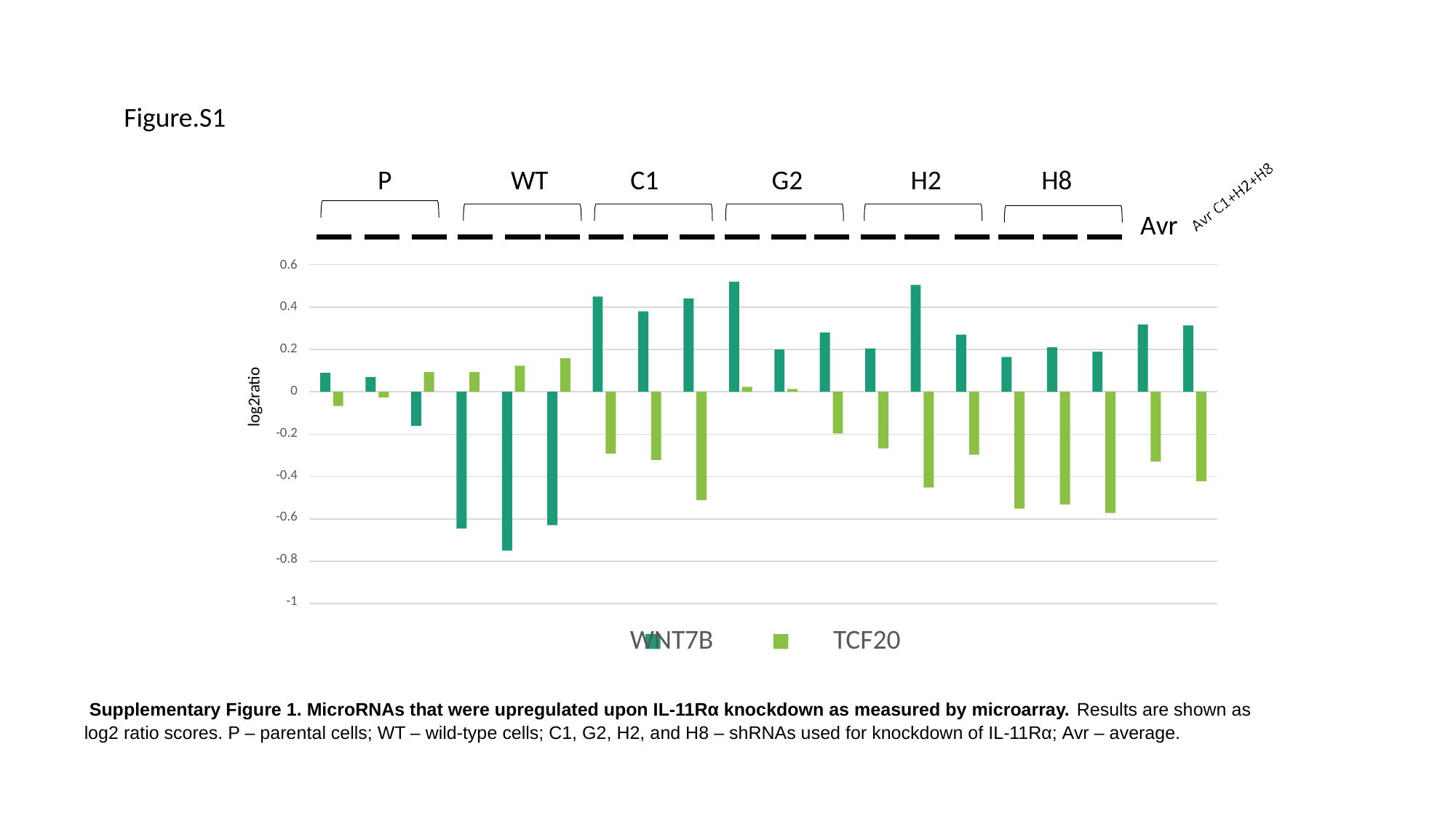

Figure.S1
P
WT
C1
G2
H2
H8
Avr
0.6
0.4
0.2
0
-0.2
-0.4
-0.6
-0.8
-1
log2ratio
WNT7B	TCF20
 Supplementary Figure 1. MicroRNAs that were upregulated upon IL-11Rα knockdown as measured by microarray. Results are shown as log2 ratio scores. P – parental cells; WT – wild-type cells; C1, G2, H2, and H8 – shRNAs used for knockdown of IL-11Rα; Avr – average.
