# Supplementary material for: Targeting IL-11R/EZH2 signaling axis as a therapeutic strategy for osteosarcoma lung metastases
Source: Discov Oncol. 2024 Jun 18;15:232. doi: 10.1007/s12672-024-01056-3 (PMC11183017; doi:10.1007/s12672-024-01056-3)
Supplement: Supplementary file 1 — Supplementary material 1. [file 12672_2024_1056_MOESM1_ESM.zip › 12672_2024_1056_MOESM1_ESM/New folder/Suppl. Fig.2.pptx]

## Slide 1
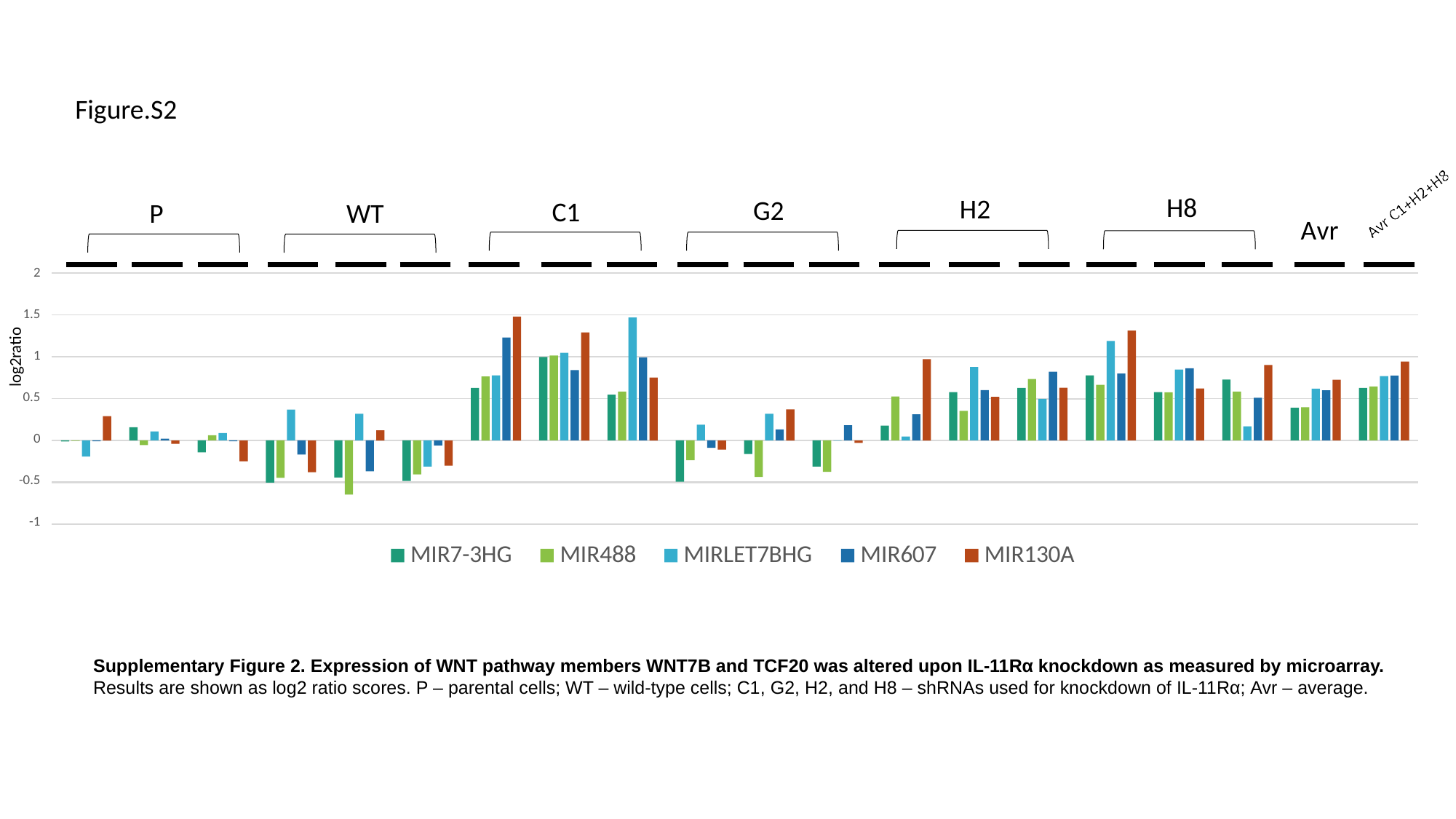

Figure.S2
H8
H2
G2
C1
P
WT
Avr
2
1.5
1
0.5
0
-0.5
-1
log2ratio
MIR7-3HG
MIR488
MIRLET7BHG
MIR607
MIR130A
Supplementary Figure 2. Expression of WNT pathway members WNT7B and TCF20 was altered upon IL-11Rα knockdown as measured by microarray.
Results are shown as log2 ratio scores. P – parental cells; WT – wild-type cells; C1, G2, H2, and H8 – shRNAs used for knockdown of IL-11Rα; Avr – average.
