# Supplementary material for: Targeting IL-11R/EZH2 signaling axis as a therapeutic strategy for osteosarcoma lung metastases
Source: Discov Oncol. 2024 Jun 18;15:232. doi: 10.1007/s12672-024-01056-3 (PMC11183017; doi:10.1007/s12672-024-01056-3)
Supplement: Supplementary file 1 — Supplementary material 1. [file 12672_2024_1056_MOESM1_ESM.zip › 12672_2024_1056_MOESM1_ESM/New folder/Suppl. Fig.3.pptx]

## Slide 1
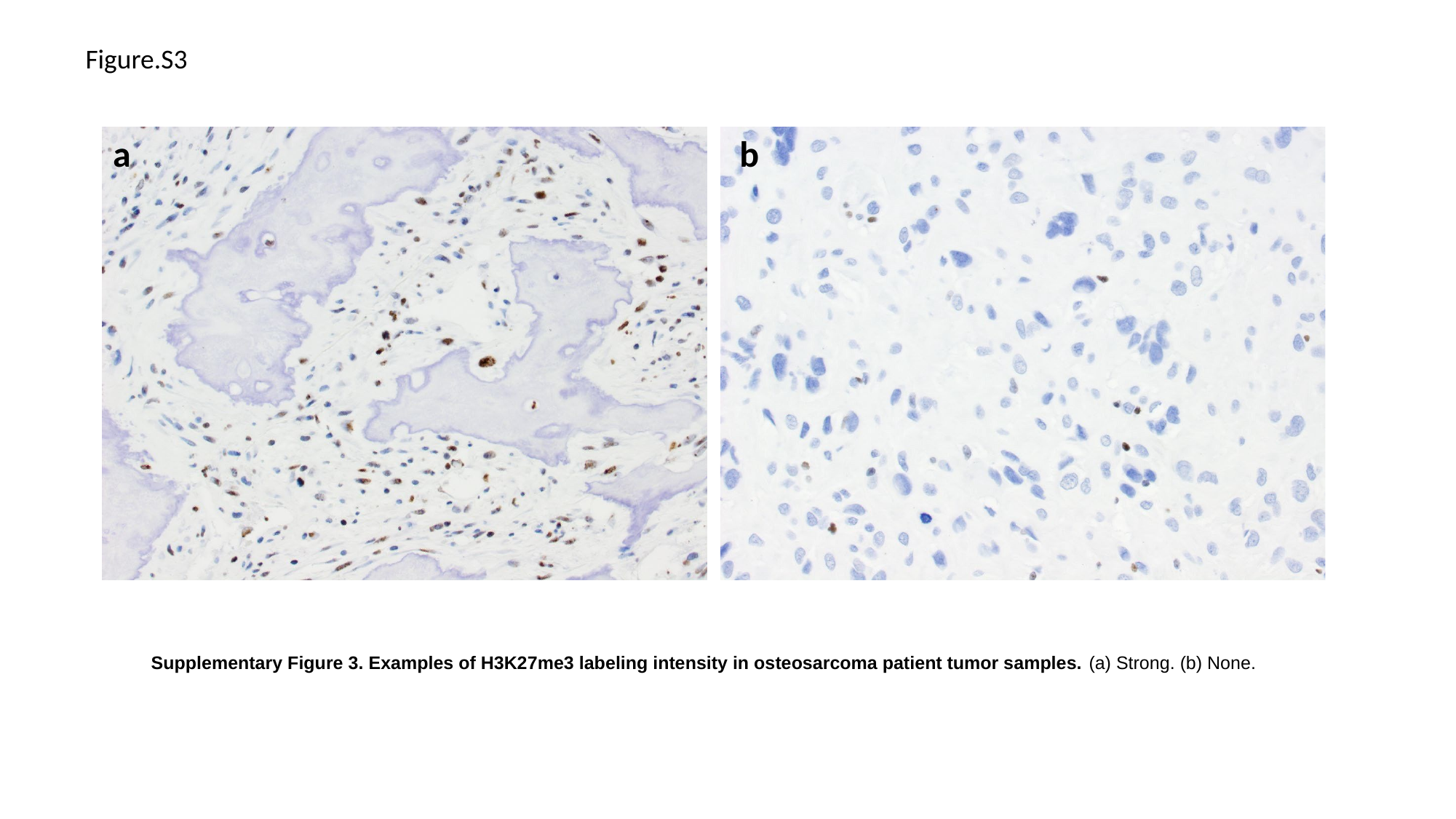

Figure.S3
a
b
Supplementary Figure 3. Examples of H3K27me3 labeling intensity in osteosarcoma patient tumor samples. (a) Strong. (b) None.
