# Supplementary material for: Targeting IL-11R/EZH2 signaling axis as a therapeutic strategy for osteosarcoma lung metastases
Source: Discov Oncol. 2024 Jun 18;15:232. doi: 10.1007/s12672-024-01056-3 (PMC11183017; doi:10.1007/s12672-024-01056-3)
Supplement: Supplementary file 1 — Supplementary material 1. [file 12672_2024_1056_MOESM1_ESM.zip › 12672_2024_1056_MOESM1_ESM/New folder/Suppl. Fig.4.pptx]

## Slide 1
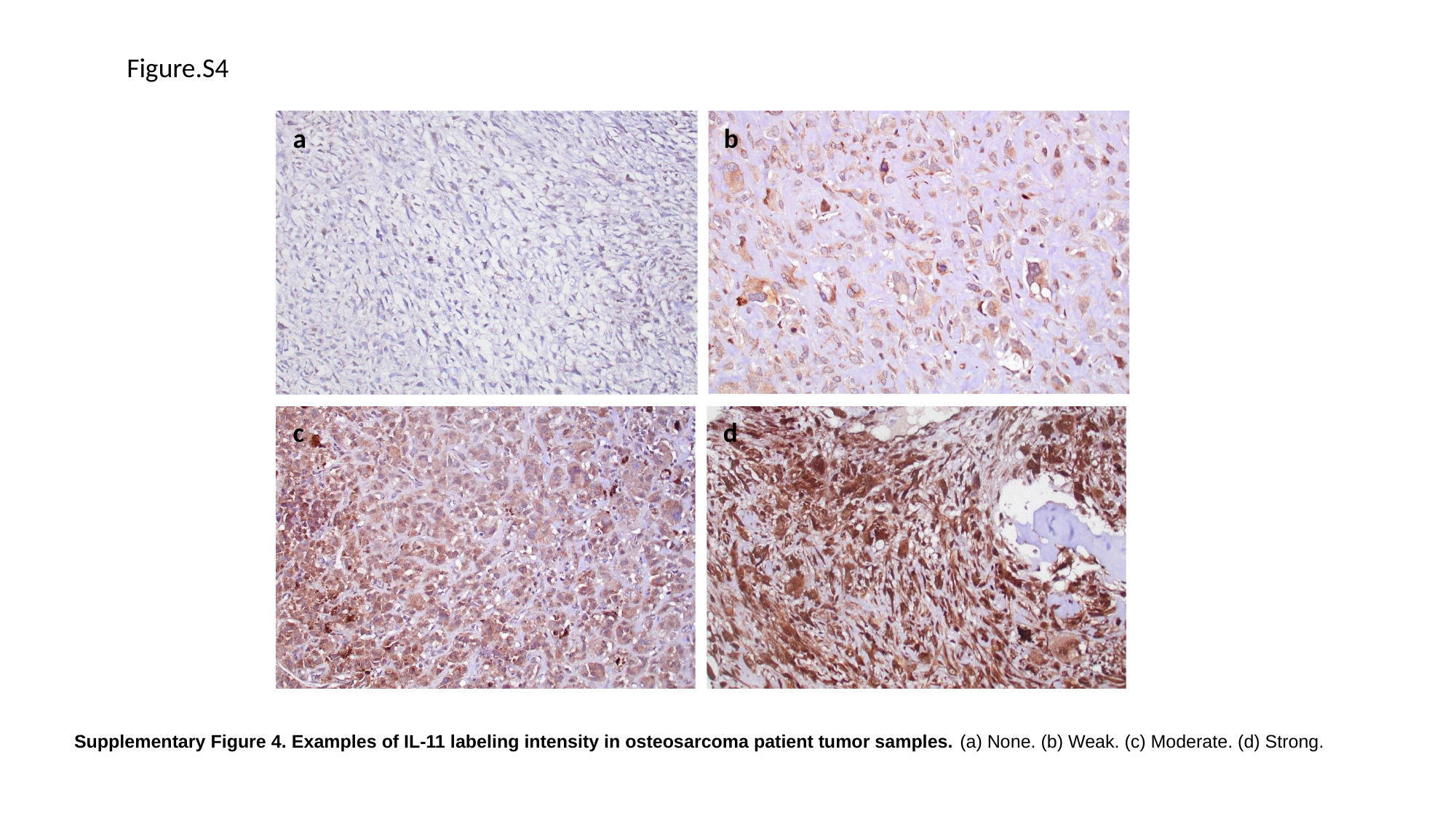

Figure.S4
a
b
c
d
Supplementary Figure 4. Examples of IL-11 labeling intensity in osteosarcoma patient tumor samples. (a) None. (b) Weak. (c) Moderate. (d) Strong.
